# Dendritic Cells: Cellular Mediators for Immunological Tolerance

**DOI:** 10.1155/2013/972865

**Published:** 2013-05-15

**Authors:** Chun Yuen J. Chung, Dirk Ysebaert, Zwi N. Berneman, Nathalie Cools

**Affiliations:** ^1^Laboratory of Experimental Hematology, Vaccine and Infectious Disease Institute (Vaxinfectio), Faculty of Medicine and Health Sciences, University of Antwerp, Antwerp University Hospital (UZA), Wilrijkstraat 10, 2650 Edegem, Belgium; ^2^Department of Hepatobiliary, Transplantation, and Endocrine Surgery, Faculty of Medicine and Health Sciences, University of Antwerp, Antwerp University Hospital (UZA), Wilrijkstraat 10, 2650 Edegem, Belgium; ^3^Center for Cell Therapy and Regenerative Medicine, Antwerp University Hospital (UZA), Wilrijkstraat 10, 2650 Edegem, Belgium

## Abstract

In general, immunological tolerance is acquired upon treatment with non-specific immunosuppressive drugs. This indiscriminate immunosuppression of the patient often causes serious side-effects, such as opportunistic infectious diseases. Therefore, the need for antigen-specific modulation of pathogenic immune responses is of crucial importance in the treatment of inflammatory diseases. In this perspective, dendritic cells (DCs) can have an important immune-regulatory function, besides their notorious antigen-presenting capacity. DCs appear to be essential for both central and peripheral tolerance. In the thymus, DCs are involved in clonal deletion of autoreactive immature T cells by presenting self-antigens. Additionally, tolerance is achieved by their interactions with T cells in the periphery and subsequent induction of T cell anergy, T cell deletion, and induction of regulatory T cells (Treg). Various studies have described, modulation of DC characteristics with the purpose to induce antigen-specific tolerance in autoimmune diseases, graft-versus-host-disease (GVHD), and transplantations. Promising results in animal models have prompted researchers to initiate first-in-men clinical trials. The purpose of current review is to provide an overview of the role of DCs in the immunopathogenesis of autoimmunity, as well as recent concepts of dendritic cell-based therapeutic opportunities in autoimmune diseases.

## 1. Introduction

Dendritic cells (DCs) are widely recognized as the most professional antigen-presenting cells (APCs). Moreover, they are indispensable in the regulation of the delicate balance between immunity and tolerance [1–3]. By interacting with other cells of the immune system through cell-cell contact or the production of cytokines, DCs induce an appropriate answer to a specific antigen. DCs can also prevent (auto)immunity by inducing apoptosis of autoreactive T cells in the thymus on the one hand (i.e., central tolerance), and by induction of anergy, deletion, or tolerance through cooperation with regulatory T cells (Treg) in the periphery on the other hand (i.e., peripheral tolerance). Consequently, it has been hypothesized that defects in the number, phenotype, and/or function of DCs cause the development of autoimmune diseases. Furthermore, DC-based antigen-specific modulation of the unwanted responses is evaluated for therapeutic approaches in recent years and may have several advantages in contrast to standard treatments which can induce a variety of complications and have serious side-effects. Indeed, considering the key role of DCs in the induction and activation of both effector T cells and Treg, DCs can be used to suppress or redirect immune responses in an antigen-specific manner. Recent investigations have shown promising results for the role of DCs as cellular treatment of autoimmune diseases and in preventing transplant rejections. Here, we discuss the role of DCs in the immunopathogenesis of autoimmunity, especially with regard to mechanisms underlying T cell tolerance, and recent concepts of DC-based therapeutic opportunities in autoimmune diseases.

## 2. Dendritic Cells: Key Regulators of Immunity and Tolerance

### 2.1. DC Subsets and Differentiation Stages

DCs originate from CD34+ hematopoietic progenitor cells in the bone marrow and are generally classified in two groups: myeloid or classical DCs (cDCs) and plasmacytoid DCs (pDCs) [1, 4]. pDCs are characterized by expression of CD123 and a high production of type I interferon (IFN). Whereas pDCs differentiate from lymphoid progenitor cells in lymphoid organs, cDCs are derived from myeloid progenitor cells in the bone marrow and differentiate into immature DCs (iDCs) with different features. (i) Langerhans cells are characterized by expression of CD11c and CD1a. Once they enter the blood circulation, they migrate to the epidermis. (ii) Interstitial DCs are CD11c+CD1a− and are found in the interstitium of various organs including the lungs, the gastrointestinal tract, afferent lymphatic vessels, and the dermis. (iii) During physiological stress, monocyte-derived DCs can originate from CD14+ monocytes under the influence of a combination of stimuli, such as granulocyte-macrophage colony-stimulating factor (GM-CSF), tumor necrosis factor-*α* (TNF-*α*), and interleukin (IL)-4. 

The widespread distribution of DCs underlines their sentinel function. Indeed, DCs are most concentrated in places of the body where invasion of pathogens is most likely. Additionally, they are also present in organs such as the heart and kidneys and lymphoid structures, including the spleen, lymph nodes, and the thymus. Where present, iDCs take up both foreign as well as self-proteins and structures and process them intracellularly to antigens that are subsequently presented in the context of major histocompatibility (MHC) class I and II molecules on the cell's surface. Once DCs capture these antigens in the presence of so-called “danger signals,” DCs undergo a complex maturation process. For this, DCs are equipped with pathogen-recognition receptors (PRRs) which detect foreign antigens (i.e., pathogen-associated molecular patterns, PAMPs) thereby activating specific signalling pathways to drive biological and immunological responses. These stimuli can be bacterial products, such as lipopolysaccharide (LPS), or viral products, including double-stranded RNA, but also proinflammatory cytokines like TNF-*α* [1, 5]. Upon maturation, DCs efficiently present the antigen/MHC complex in combination with co-stimulatory molecules, have changed their pattern of cytokine production [6], and will migrate to the lymph nodes where they eventually activate T cells [1, 7].

### 2.2. The Immunological Synapse

DCs bridge innate and adaptive immunity, integrate a variety of stimuli, and establish protective immunity. For this, efficient communication between DCs and T cells is warranted and must take place in the presence of at least 3 signals. First, the presented antigen/MHC complex must bind with the T cell receptor (TCR) of T cells (i.e., “signal 1”). Second, costimulation is obligatory for T cell activation (i.e., “signal 2”). For instance, binding of CD80/86 molecules on DCs with CD28 present on the cell membrane of T cells results in T cell stimulation. For a long time, it was believed that antigen recognition in the absence of co-stimulatory factors results in T cell anergy [5]. However, to date a variety of co-stimulatory pathways have been identified and are currently classified based on their impact on primed T cells [8]. Indeed, pathways delivering activatory signals to T cells are termed co-stimulatory pathways, whereas pathways delivering tolerogenic signals to T cells are termed coinhibitory pathways. Furthermore, it is generally accepted that an additional “signal 3” is also needed for efficient T cell stimulation and polarization. A well-known example is the potent induction of interferon (IFN)-*γ*-producing T helper type 1 (Th1) cells by interleukin (IL)-12 produced by DCs as response to certain microbial stimuli [6, 9]. Furthermore, both *in vitro* as well as *in vivo* studies have demonstrated that CD40 ligation of CD8+ T cells is necessary for optimal clonal expansion, effector function, and generation of a memory population [10–12]. Raveney and Morgan [13] have suggested that alterations in one of these three signals could shift the balance to tolerance or (auto)immunity. Recently, Kalinski et al. [6, 7] described a potential fourth signal delivered by DCs that results in the upregulation of chemokine receptors on effector T cells and that thus might play part in organ-specific chemotaxis of T cells. 

Depending on the cytokines present upon T cell activation, naïve CD4+ T helper (Th) cells can acquire a variety of immune effector phenotypes [14]. In brief, release of IL-12 by DCs promotes a Th type 1 (Th1) response. Th1 cells mediate a cellular as well as delayed-type hypersensibility immune response with proliferation of T cells and production of IFN-*γ* and IL-2. Furthermore, Th1 cells induce stimulation of CD8+ cytotoxic T cells (CTL). Th2 cells are stimulated through OX40 ligation by DCs, produce mainly IL-4, IL-5, and IL-13, and promote the activation of B cells, which can also be involved in autoimmunity [15]. Tumor-growth factor (TGF)-*β*, in the absence of proinflammatory cytokines, induces Tregs, while TGF-*β*, IL-1, and IL-6 are needed for induction of Th17 cells [16]. Tregs are immune suppressive, and hence counteract effector T cells. In contrast, Th17 cells generate an influx of neutrophils and cause allergic or autoimmune reactions.

### 2.3. Dendritic Cells Inducing T cell Tolerance

DCs are essential for both central and peripheral tolerance [5, 17–20]. Central tolerance occurs in the thymus where thymoid DCs present self-antigens to developing T cells. Subsequently, lymphocytes with autoreactivity above a certain threshold are deleted, a process called clonal deletion. Additionally, naturally occurring Tregs (nTregs) are positively selected by thymoid DCs in the thymus [21]. However, some limitations of central tolerance resulting in escape of potentially autoreactive T cells underlie the need for effective peripheral silencing mechanisms. In this regard, several mechanisms mediated by DCs have been proposed. (i) It has been suggested that iDCs fail to stimulate T cells sufficiently because of their low expression of MHC molecules and co-stimulatory factors. This results in T cell anergy [1, 22]. (ii) It has also been reported that suboptimal antigen presentation, together with indoleamine 2,3-dioxygenase (IDO) or Fas (CD95) expression by iDCs leads to inhibition of T cell proliferation and T cell deletion [5]. (iii) Furthermore, DCs are able to induce Tregs to preserve immune tolerance to self-antigens [17] as well as to certain foreign antigens [1, 2, 5]. Moreover, IL-10-producing regulatory type 1 T cells (Tr1) are also promoted by DCs, hereby reinforcing peripheral tolerance [17, 23, 24] (for review on Treg subsets, see [21]).

 Zehn and Bevan [19] showed that central tolerance accompanied by equal efficient peripheral tolerance is very efficient in withholding high avidity autoreactive T cells. Despite these mechanisms, some low avidity autoreactive T cells may escape and be present in the periphery. Therefore, it has been suggested that their activation can occur by cross-reaction with foreign antigens, subsequently driving T cells to differentiate into effector T cells causing autoimmunity.

## 3. Role of DCs in the Pathogenesis of Autoimmunity

A healthy immune system recognizes and eliminates invading pathogens, but preserves tolerance for self-antigens. In contrast, autoimmune diseases develop when self-antigens are recognized as foreign by the immune system, resulting in hyperactivity of both cellular and humoral immunity against these antigens. The underlying mechanisms abrogating immune tolerance for self-antigens are still unclear. However, given the central role of DCs in maintaining the balance between (auto)immunity and tolerance, they are believed to play an important role in this process [2, 25].

While neonatal mice who have undergone thymectomy [26] or thymic deletion [27] develop severe systemic autoimmune diseases, similar clinical outcomes in mice were obtained upon the depletion of both cDCs and pDCs. Indeed, Ohnmacht et al. [28] observed that constitutive ablation of DCs in mice leads to the breakdown of tolerance for self-antigens resulting in severe spontaneous autoimmune responses possibly caused by an increased amount of Th1 and Th17 cells. Moreover, a variety of antibodies against both nuclear and tissue-specific autogens was found in these mice. The authors showed that DCs with a short lifespan did not induce an efficient tolerance of CD4+ T cells, which was reflected in the thymus as a decreased negative selection and as a shortage of tolerogenic DCs in the periphery. Albeit that others demonstrated that increasing the lifespan of DCs through inhibition of apoptosis also induced autoimmunity in mice [29], thereby emphasizing the ambiguous role of DCs in immunity as well as tolerance. Of interest, it was recently described that peripheral T cells can reenter the thymus, where they target thymic DCs and medullary thymic epithelial cells. As a consequence, negative selection in the thymus was suppressed with breakthrough of T cells with a high affinity for self-antigens causing autoimmune diseases [30]. Altogether these studies underscore the importance of immune regulation in the thymus and periphery controlling (auto)immunity.

Whereas it is generally accepted that DCs in steady state, although loaded with self-antigens from their environment, do not trigger autoimmunity [5, 18, 31], discrepancies in DC number, phenotype, and function are believed to contribute to disease [2, 32–36]. Indeed, in animal models for type I diabetes, arthritis [17], Wiskott-Aldrich syndrome [20], and systemic lupus erythematosus (SLE) [37], it was shown that increased access of DCs to intracellular autogens—mediated by increased amounts of apoptotic cells or insufficient clearance of these cells—resulted in subsequent autogen presentation and activation of T cells. In an attempt to elucidate possible underlying mechanisms, Sawatani et al. [29] attributed a role in the phagocytic activity and antigen-presenting function of DCs to the dendritic cell-specific transmembrane protein (DC-STAMP). Indeed, in DC-STAMP-deficient mice the authors found increased *in vitro* phagocytosis and antigen presentation by DCs which could give rise to systemic autoimmunity [29]. Because of the high expression of MHC class II and co-stimulatory molecules, mature DCs are utmost equipped to activate T cells. In addition, both mature cDC and pDC produce proinflammatory cytokines, including IL-12p70 and type 1 IFN, respectively, which could contribute to the pathogenesis of autoimmunity [38, 39]. In this perspective, Lech et al. [40] demonstrated that the absence of the Sigirr gene, which is a variant of Toll-like receptor (TLR)/interleukin 1 receptor (Tir) family and suppresses the TLR-mediated pathogen recognition in DCs, resulted in enhanced activation of DCs. This was evidenced by increased expression of proinflammatory mediators and was associated with the development of murine lupus. In inflamed tissues, such as the synovium in rheumatoid arthritis (RA), these proinflammatory signalling molecules are found in high amounts in DCs in the vicinity of T cells. For this, it has been hypothesized that DCs maintain the local autoreactive T cell response [38]. Besides, a correlation exists between the amount of DCs and the concentration of anticitrullinated peptide antibodies in serum of RA patients [38], suggesting a possible regulatory role for DCs in the production of autoantibodies in RA. Furthermore, DCs are described to enhance the formation of ectopic lymphoid tissues in target organs. The underlying mechanism is probably explained by chemotactic cytokines released by DCs leading to lymphoid neogenesis and recruitment of leukocytes in the inflamed tissue, including the synovium [41] and the pancreatic islets [42]. In other studies the formation of ectopic lymphoid structures was ascribed to B cells [16]. DCs can also directly damage surrounding tissues. In this perspective, it was recently shown that monocyte-derived DCs could destroy the cartilage in joints through the production of TNF-*α* [43].

## 4. Tolerogenic DCs-Based Treatments

Efforts to bring DC vaccination to the clinic aiming induction of tolerance, were initiated by Dhodapkar et al. who demonstrated that pulsing immature DCs with influenza matrix protein (IMP) and keyhole limpet hemocyanin (KLH) resulted in a decrease of influenza-specific CD8+ IFN-*γ*-secreting T cells, while peptide-specific IL-10-secreting T cells appeared [44]. Menges et al. [45] showed in mice that bone marrow-derived DCs treated with TNF-*α*, so-called semi-mature DCs, were able to suppress the course of experimental autoimmune encephalomyelitis (EAE), the animal model for MS, through the activation of IL-10-secreting Tregs. Unfortunately, the semi-mature phenotype of these DCs is not stable since they produce proinflammatory cytokines upon introduction of a secondary stimulus (e.g., LPS). In contrast, biological molecules and pharmaceutical agents, including vitamin D_3_, IL-10, the corticosteroid dexamethasone, and the immunosuppressive drug rapamycine, are known to induce immature DCs with a low immunogenic character, that is, no upregulation of co-stimulatory molecules or secretion of proinflammatory cytokines, so-called tolerogenic DCs (tolDCs). Indeed, treatment of DCs with vitamin D_3_ or equivalents resulted in an increased release of IL-10, whereas the expression of co-stimulatory molecules and bioactive IL-12 was downregulated. Moreover, the authors demonstrated that these tolDCs induced tolerance to the allograft in a mouse model [46]. Another example is triptolide, derived from a Chinese herb, which was found to have potent immunosuppressive effects as demonstrated by its prevention of DC migration and release of chemokines as well as subsequent inhibition of T cell activation and proliferation [47, 48]. Treatment of human DCs with the immunoregulatory neuropeptide, vasoactive intestinal peptide (VIP), induces significant production of anti-inflammatory cytokines, such as IL-10, causes a decrease in the expression of the co-stimulatory molecules CD80/86, and inhibits the phagocytic activity by DCs [49, 50]. Importantly, these DCs_VIP_ keep their immature phenotype after exposure to inflammatory signals like TNF-*α* and LPS. Hence, a stable immature phenotype is generated. In addition, a population of antigen-specific Tr1-like cells, producing both IL-10 and TGF-*β* and inhibiting the proliferation of Th1 cells, was found. Moreover, CD8+CD28− Tregs were also induced contributing to the antigen-specific tolerance. Vaccination of DCs_VIP_ in mice during development of collagen-induced arthritis (CIA), EAE, and graft-versus-host disease (GVHD) in allogeneic bone marrow transplantation induced organ-specific tolerance and suppressed the course of disease. 

Recently, genetic engineering has made its way in the quest for therapeutic possibilities for autoimmune diseases. Indeed, the insertion of new DNA in order to enhance tolDC function has been investigated. For example, by transfection of DNA coding for the Fas-ligand [51] or TNF-related apoptosis-inducing ligand (TRAIL) so-called “killer” DCs could be obtained. These genetically modified DCs efficiently induce T cell apoptosis, suppress autoimmune arthritis, and prevent rejection of donor-specific heart transplants in animal models [52]. In addition, injections of genetically modified IL-4-producing DCs in CIA suppress the development and inflammation level of arthritis. In a study of Kaneko et al. [53], however, these DCs caused an accelerated immune reaction and rejection of the allograft, making these IL-4-producing DCs less attractive for therapeutic use. Alternatively, selective knockout of the expression of DC-characteristic molecules and functions has been intensively investigated. Utilizing RNA interference (RNAi) directed at IL-12p35 in order to generate IL-12-silenced DCs resulted in the prolongation of the intestinal allograft lifespan in rats [54]. Similar results were achieved in animal models after silencing of RelB and NF-*κ*B which resulted in allogeneic donor-specific hyporesponsitivity of the T cells, associated with an inhibition of the cytokine production of Th1 cells, and prolonged survival of the cardiac allograft in mice [55]. Recently, a clinical trial administering monocyte-derived DCs genetically modified with antisense oligonucleotides targeting the transcripts of CD40, CD80, and CD86, thereby selectively reducing their surface expression [56], was performed in type 1 diabetes patients and was proven to be safe, well tolerated, and without any adverse effects [57]. Whether recently identified negative regulators of DC activation, including zDC [58] and FOXO3 [59], hold promise for future DC-based tolerance-inducing strategies remains to be established.

## 5. Induction of Long-Lasting Immune Tolerance

Ideally, therapies for immunosuppression must also be durable. This means that the ability to regulate the autoimmune response has to be permanent or at least for many years following intervention, for instance, via the generation of self-antigen-specific Tregs.

Indeed, different *in vitro* generated tolDCs, including IL-10-modulated DCs [60] and DCs treated with a combination of dexamethasone and 1*α*,25-dihydroxyvitamin D_3_ [61], were shown to induce Tregs. In addition, Housley et al. [62] demonstrated that activation of PPAR*γ*, a nuclear hormone receptor, in CD103+ DCs from the gut-associated lymphoid tissue (GALT) in mice was important for the regulation of retinoic acid secretion and Treg generation by DCs. This might contribute to the suppression of autoimmunity since other studies [63, 64] reported that CD103+ GALT DCs induce an increased conversion of effector T cells to Tregs in a retinoic acid-dependent manner. Interestingly, some tolDC populations also promote the induction of regulatory B cells (Bregs), underlining suitability for tolerance-inducing strategies [61].

Whereas DCs drive the differentiation of Tregs in order to control immune responses, Tregs also modulate DC phenotype and function [65]. Indeed, Gabryšová et al. [66] showed that the autoimmune response was limited by a negative feedback system started by the antigen-induced differentiation of Th1 cells into IL-10-producing Tregs which on their turn inhibited DC maturation, thereby suppressing Th1 responses and completing the negative feedback loop. Furthermore, following depletion of FoxP3+ T cells, DCs that lack the expression of MHC class II molecules were not able to make cognate interactions with CD4+ T cells resulting in spontaneous and fatal CTL-mediated autoimmunity, indicating the critical suppressive role of the FoxP3+ Treg population in maintaining DCs in a tolerogenic state [67]. Overall, these findings highlight the importance of the bidirectional crosstalk between DCs and Tregs in maintaining and inducing tolerance.

## 6. Discussion

The use of tolerogenic DCs as cellular mediators for the induction of tolerance in autoimmune diseases and transplantation is very promising and could in the future complement or even substitute immunosuppressive agents which have important side effects including increased risk of infections. However, some open-standing questions need to be addressed before DC-based vaccines could be implemented in the clinic [68].

A first challenge is the identification of a maturation-resistant subtype of DCs. For instance, while CD8*α*+ DCs, the mouse equivalents of human myeloid DCs can act tolerogenic by inducing T cell apoptosis via their expression of Fas-ligands [69, 70], others demonstrated that these CD8*α*+ DCs released high amounts of IL-12 and were able to stimulate CD8+ CTL [71]. Additionally, Waithman et al. [72] described a CD11c+CD207+ skin-derived DC subset presenting self-antigens in the draining lymph nodes and inducing deletion of MHC class I-restricted autoreactive T cells, thereby contributing to tolerance. In contrast, others showed that these skin-derived DCs drive autoimmune tissue destruction. Hence, tolDCs cannot solely be distinguished based on their phenotype but must be carefully investigated regarding their stability and tolerogenic effect, especially after vaccination. Given the risk of *in vivo* reactivation, this is particularly of importance in any pathological state with an underlying inflammatory microenvironment.

Ideally, therapies for immunosuppression must also be (self-) antigen specific and durable. In this respect, Hawiger et al. [73] devised a DC-targeting system. Using a monoclonal antibody targeting DEC-205, a DC-restricted endocytic receptor, the authors delivered a specific antigen to DC. Albeit that initially an extensive T cell proliferation was observed, this was followed by T cell anergy and deletion. With these results, the authors suggested a possible role for inducing antigen-specific peripheral tolerance with this system. Unfortunately, in combination with a DC maturation stimulus, this strategy resulted in immune activation, thereby limiting its clinical use for the treatment of autoimmunity. Hence better insights in the role of distinct DC populations are warranted. In this respect, antigens delivered via antibodies to CLEC9A, a recently discovered C-type lectin receptor which is selectively expressed by CD141+ myeloid DCs, were shown to be a promising strategy to efficiently induce immunity against infections and malignant diseases [74, 75]. Likewise, antigens specifically delivered to migratory DCs, trafficking from peripheral tissues to draining lymph nodes charged with self-antigens, were shown to be superior in generating Tregs *in vivo* and consequently drastically improved the outcome of autoimmune disease [76]. In addition, durable tolerance means that the ability to regulate the autoimmune response has to be permanent or at least for many years following intervention, for instance, via the generation of self-antigen-specific Tregs. For this, increased knowledge with regard to the pharmacokinetic and pharmacodynamic properties of DC-based strategies is imperative. Other related questions that need to be taken into consideration for the success of this approach are the timing of DC therapy (e.g., a prophylactic or a therapeutic treatment regimen) and selection of antigenic peptide(s) for loading DCs. Additionally, parameters such as antigen dose, number of cells, requirements for repetitive DC vaccinations, and the route of administration need to be addressed in clinical application. Finally, ethical issues may also arise, especially with regard to the implementation of experimental therapy for graft acceptance upon transplantation while there is a shortage of organ donations. Note must be taken that patient-specific treatment modalities, including DC-based vaccination, are very expensive and require careful monitoring of treatment-related efficacy and toxicity, individual patient morbidity, and quality of life, as well as societal costs. 
